# The determinants of 5th minute low Apgar score among newborns who delivered at public hospitals in Hawassa City, South Ethiopia

**DOI:** 10.1186/s12887-021-02745-6

**Published:** 2021-06-08

**Authors:** Alex Yeshaneh, Andargachew Kassa, Zemenu Yohannes Kassa, Daniel Adane, Yohannes Fikadu, Semahegn Tilahun Wassie, Biresaw Wassihun Alemu, Mesfin Tadese, Solomon Shitu, Haimanot Abebe

**Affiliations:** 1 Department of Midwifery, College of Medicine and Health Sciences, Wolkite University, Wolkite, Ethiopia; 2 Department of Nursing, College of Medicine and Health Sciences, Hawassa University, Hawassa, Ethiopia; 3 Department of midwifery, College of Medicine and Health Sciences, Hawassa University, Hawassa, Ethiopia; 4 Department of Midwifery, College of Medicine and Health Sciences, Mizan Tepi University, Mizan Tepi, Ethiopia; 5 Department of Midwifery, College of Medicine and Health Sciences, Arbaminch University, Arbaminch, Ethiopia; 6 Department of Midwifery, College of Medicine and Health Sciences, Debrebirhan University, Debrebirhan, Ethiopia; 7 Department of Public Health, College of Medicine and Health Sciences, Wolkite University, Wolkite, Ethiopia

**Keywords:** 5th minute, Newborn, Apgar score, Determinant, Ethiopia

## Abstract

**Background:**

Newborn morbidity and mortality are forecasted using the Apgar scores. Obstetricians worldwide have used the Apgar score for more than half a century for the assessment of immediate newborn conditions. It is a simple and convenient evaluation system that offers a standardized and effective assessment of newborn infants. Neonatal morbidity and mortality can be reduced if high-risk neonates are identified and managed adequately. This study aimed to assess the determinants of 5th minute low Apgar score among newborns at Public hospitals in Hawassa city, South Ethiopia.

**Methods:**

A hospital-based unmatched case-control study was conducted at Public Hospitals in Hawassa city. Data were collected from 134 cases and 267 controls using a structured and pre-tested questionnaire by observing, interviewing, and reviewing patient cards. Newborns who delivered with a 5th minute Apgar score < 7 were considered as cases; whereas a similar group of newborns with a 5th minute Apgar score of ≥ 7 were categorized as controls. A consecutive sampling technique was employed to recruit cases, while a simple random sampling technique was used to select controls. Data entry and analysis were performed using Epi Data version 3.1 and SPSS version 20 respectively. Binary and multivariable analyses with a 95 % confidence level were performed. In the final model, variables with *P* < 0.05 were considered statistically significant.

**Results:**

After controlling for possible confounding factors, the results showed that lack of physical and emotional support during labor and delivery [AOR = 3.5, 95 %CI:1.82–6.76], rural residence [AOR = 4, 95 %CI: 2.21–7.34], lack of antenatal care follow up [AOR = 3.5, 95 % CI: 1.91–6.33], anemia during pregnancy [AOR = 2.3,95 %CI: 1.10–4.71] and low birth weight [AOR = 6.2, 95 %CI: 2.78–14.03] were determinant factors of low Apgar scores. The area under the Apgar score ROC curve was 87.4 %.

**Conclusions:**

Lack of physical and emotional support, rural residence, lack of ANC follow-up, low birth weight, and anemia during pregnancy were determinant factors of a low Apgar score. `Effective health education during preconception about anemia during pregnancy and ANC will help in detecting high-risk pregnancies that lead to a low Apgar score. In addition to the standard care of using electronic fetal monitoring, increasing access to compassion ships during labor and delivery is recommended.

## Introduction

The neonatal period refers to birth to the first 28 days of life and the most perilous period of life because of the numerous challenges that the neonate faces. The risk of mortality is the highest during this period of life [1]. The APGAR is a general and quick assessment of newborn well-being immediately after birth and is recorded at one minute and five minutes from the time of birth [2]. The Apgar scoring system offers a consistent assessment of infants after delivery [3]. The one minute Apgar score indicates the baby’s physical health and to determine whether immediate or future medical treatment will be required. The five-minute Apgar score assesses how the baby has reacted to previous resuscitation attempts if such efforts were made [2].

The score ranges from 0 to 2 for each of the five characteristics with a maximum final total score of ten (See Table 1 below). At the 1 min APGAR, scores between 7 and 10 indicate that the baby will need only routine post-delivery care. Scores between 4 and 6 indicate that some assistance for breathing might be required. Scores under four can call for prompt measures. A five minute Apgar score of 7–10 is normal. If the score is below seven, the baby will continue to be monitored and re-evaluated every 5 min for up to 20 min. Thus, the Apgar score system has a continuing value for predicting neonatal and post-neonatal adverse outcomes and is applicable to singleton and twin pregnancies and in various race/ethnic groups [2, 4].

**Table 1:** How numerical ratings are assigned to describe the five Apgar signs and symptoms used to assess a baby’s condition at birth

Apgar sign	0	1	2
**Activity** (muscle tone)	Limp, no movement, muscles are floppy and loose	Some muscle tone, some flexing of arms and legs	Active, spontaneous motion, flexed arms and legs that resist extension
**Pulse** (heart rate)	No heart rate	Fewer than 100 beats per minute	At least 100 beats per minute
**Grimace** (reflexes)	No response to stimulation	Facial grimace during stimulation	Pulls away, coughs, cries vigorously, or sneezes during stimulation
**Appearance**	Entire body is blue or pale	Good color in body with bluish hands or feet	Good color all over
**Respiration** (breathing)	Not breathing	Slow or irregular breathing, weak cry, whimpering	Normal rate and effort of breathing; good, strong cry

Globally, nearly 7,000 newborns die every day at a rate of 19 per 1,000 live births [5] and an estimated 23 % of neonatal deaths and 10 % of all deaths in under-five children occur as a result of the low Apgar score [6]. Birth asphyxia is a global problem, especially in developing countries and the severity of asphyxia is widely assessed using the Apgar score. Worldwide, 4 million deaths occur annually due to birth asphyxia, representing 38 % of all deaths in children under five years of age [7, 8]. In developing countries, neonatal mortality and morbidity due to low Apgar scores account for 100–250/1000 live births compared to 5–10/1000 live births in developed countries [9]. In Ethiopia, the neonatal mortality rate is 29 deaths per 1,000 live births [10]. Besides, in Gondar Ethiopia, 13.8 % of neonates had a low Apgar score at the 5th minute of life and among these 22.2 % died within the neonatal period [11].

If the world goes through with the current pace, 28 million newborns would die between 2018 and 2030, and the majority of these deaths would occur in sub-Saharan Africa and South Asia [12]. Reducing neonatal mortality is important not only to reduce the proportions of under-five mortality but also the health interventions needed to address the major causes of neonatal deaths generally differ from those needed to address other under-five deaths [5]. Reducing the low Apgar score has an important contribution to the achievement of SDG in reducing neonatal mortality.

A neonate’s continued capacity to survive and thrive can be shown by five minutes Apgar score [4]. If there is a change in recovery rates between a few minutes of newborn life, significant clinical information can be detected. Furthermore, it provides acumen into health system issues in the intrapartum period. Understanding the factors associated with the transition between the 1st minute Apgar score and 5th minute Apgar score has the potential to provide time for intervention or further investigation [13]. For this reason, the Apgar score at 5 min is a better forecaster of later outcomes than the Apgar score at 1 min [4].

Available evidences have shown that low Apgar scores increase the risk of neonatal and infant death and neurologic disability including cerebral palsy, epilepsy, cognitive impairment, and low academic performance [14, 15]. Prematurity, congenital malformations, maternal analgesia, trauma, and inter-observer variability are independent determinant factors of low Apgar scores [2].

Even though much has been done to prevent neonatal morbidity and mortality, still, the levels of neonatal mortality in sub-Saharan African countries were significant. One of the early neonatal assessment tools for neonatal status is the Apgar score. Therefore, by identifying the determinant factors of the low Apgar score the preventive action could be taken to reduce the number of neonatal deaths and morbidity associated with a low Apgar score. Published studies in Ethiopia that identified the determinant factors of the 5th minute Apgar score was limited [11, 16, 17]. Therefore, this study aimed to identify significant determinant factors of low Apgar scores among newborns in Hawassa city, South Ethiopia (Table 1).

## Methods and materials

### Study design and Area

A hospital-based unmatched case-control study with a control-to-case ratio of 2:1 was conducted among newborns delivered at Public hospitals in Hawassa city from March 20 to August 30/2018. Hawassa city is the southern nations, nationalities, and peoples’ region capital located 273 km from Addis Ababa and 157,879 people live in this city according to the 2007 census. Hawassa university’s comprehensive specialized hospital (HUCSH) and Adare general hospital are two public hospitals found in Hawassa city. Ten obstetricians and gynecologists, 74 midwives, 2 nurses, 4 integrated emergency surgeons, interns’ students, and residents of obstetrics and gynecology departments staffed the obstetric ward of these hospitals. There was an average of 35 deliveries per day/1043 deliveries per month in both hospitals.

### Study participants and eligibility criteria

Singleton term and post-term live births in the two public hospitals during the study period, irrespective of the mode of delivery, were considered eligible for the study. The Apgar score of every child was assessed and newborns who delivered with 5th minute Apgar score < 7 were considered as cases; whereas a similar group of newborns with 5th minute Apgar score ≥ 7 were categorized as controls. Newborns that were referred from other health institutions, gross congenital anomalies incompatible with life (Such as anencephaly, severe hydrocephalus, and gastroschisis) and multiple pregnancies were excluded. Because these conditions are known risk factors for a low Apgar score.

### Sample size determination

The Sample sizes were calculated using Open Epi statistical software 2nd edition [16] by considering 80 % power of the study, 95 % confidence level, and control-to-case ratio of 2:1and the proportion of exposure among controls and cases of different variables which were suggested by the obstetrician, most commonly cited in the literature as a determinant factor of a low Apgar score, variables with lower effect size (OR) which gives a larger sample size and those variables in which authors intended to test in the study. From the alternative sample sizes, by considering a 10 % non-response rate, the largest sample size (401; 134 cases and 267 controls) was selected (Table 2).

**Table 2:** Sample size calculation for unmatched case-control study by open Epi software [18]

No	Variables	Proportion of controls with exposure	Proportion of cases with exposure	95 %CI OR	Sample size	Ref
1.	Meconium stained liquor	25.1 %	40.13 %	2(1.2–2.9)	Cases: 122	[19]
					Control: 243	
					**Total: 365**	
2.	Operative delivery	45.7 %	62.73 %	2(1.02–3.11)	Cases: 109	[20]
					Control: 218	
					Total:327	
3.	Induction/augmentation	59.3 %	87.6 %	4.85(1.71,13)	**Cases:34**	[11]
					**Control:68** **Total:102**	

### Sampling technique and procedure

First, the data collectors measured the Apgar scores of all newborns during the data collection period. Then, a consecutive sampling technique was employed to recruit cases, while a simple random sampling technique was used to select controls.

### Data collection tools and procedures

The data were collected by interviewing the mothers, reviewing medical records, measuring the weight, and assessing the Apgar scores of the newborns. Five trained midwives working in the delivery wards of the two hospitals collected the data using a structured and pretested questionnaire prepared in the Amharic language. Eligible mothers were interviewed face to face within 24 h after delivery for socio-demographic information. The medical records of the mothers were reviewed and relevant information including the last-normal menstrual period and ultrasound dating of pregnancy were extracted from the questionnaire.

Using a calibrated Seca scale the weight of the newborns was measured immediately after birth. To address the design, the data collectors were assigned an eight hour shift every day. Each newborn recruited was assessed for Apgar scores in the 1st and 5th minutes. The Apgar score was estimated using five variables and then the score of each finding was summed up.

### Data collectors

Data collectors were five BSc midwives (Three from Adare General Hospital and Two from HUCSH) working in two public hospitals. In these two public hospitals, a midwife has been working an 8 h shift. Three of those who work in Adare General Hospital collected data from the Hawassa University Comprehensive Specialized Hospital (HUCSH) in 8 h shift when they were duty-free from Adare General Hospital. One midwife at a time went to HUCSH to collect data for 8 h and then on, she/he handover for the next data collectors remaining next 8 h and last 8 h. Those who work in HUCSH also went to Adare General Hospital in the same fashion as those who work in Adare General Hospital.

### Data quality assurance and control

One day of training was given for data collectors and supervisors on objectives and the standard procedures of Apgar score estimation. Demonstrations were held which was aided by video and doll. By considering 5 % of the total sample size, a pretest was conducted one week before the start of actual data collection in the hospital which was not part of this study. Then, the questionnaire was assessed for its clarity, length, completeness and the necessary correction was done accordingly.

Throughout the data collection, interviewers were supervised, regular meetings were held between the data collectors and the principal investigator together in which problematic issues arising from interviews during the data collection and any challenges found were discussed. The completeness of the data was evaluated by MSc clinical midwifery students daily. The collected data were again reviewed and checked for its completeness before data entry. The data entry format template was prepared and programmed by the principal investigator.

### Data management and analysis

Data were coded, cleaned, and entered into Epi Data version 3.1 and exported to SPSS version 20.0 for statistical analysis. The outcome variable was coded as ‘1’ for low Apgar (cases) and ‘0’ for normal Apgar score (controls). Descriptive statistical analysis was carried out to compute frequency, percentage, and mean for continuous independent variables. The socio-demographic and other background profiles of the cases and controls were compared using the chi-square test. Before the analysis, the assumptions of the chi-square test were checked. When smaller expected frequencies were encountered, re-categorization of variables or merger of the levels was made.

Binary logistic regression analysis was used to ascertain the association between explanatory and outcome variables and some of the statistical tests like the odds ratio was used. Variables with a significant association at *P* < 0.25 [20] in the binary analysis were entered into multivariable analysis using the enter method to determine the determinant factors of the low Apgar score and those variables *P* < 0.05 were considered to be statistically significant. Hosmer- Lemeshow tests for goodness of fit were carried out and found to be good fit (*p* = 0.889(> 0.05)). Finally, the results were presented in texts, tables, and graphs and it was discussed using the odds ratio and 95 % confidence level.

### Ethical approval and consent to participate

 Ethical clearance was obtained from Hawassa University College of medicine and health science, an institutional review board (Ref.No:IRB/172/10). Permission to conduct the study was also obtained from Adare General Hospital and HUCSH. Participants were informed about the purpose and objective of the study. They were also informed that they have the right to discontinue or refuse to participate in the study if they were not comfortable with the questionnaire. Informed written consent was obtained from each study participant. Confidentiality of information and privacy has been observed.

### Statement of confirmation

 All methods aforementioned above were carried out in accordance with relevant guidelines and regulations.

## Result

### Socio-demographic characteristics of study participants

A total of 134 cases and 267 controls were studied. From the total of 401mother-newborn dyads who were invited for an interview, all of them were approached in the study. The mean (± SD) age of the mothers was 27.4(± 3.2) years. About three fourth of the age distribution of mothers of cases and controls (75.4 and 78.3 %, respectively) were in the age group between 20 and 35 years. As shown by the independent sample t-test (*P*-value = 0.768), the mean age difference between cases and controls is not statistically significant. Proportion of mothers of cases who were housewives was significantly higher than that of controls (57.5 % vs. 40.4 %, *p* = 0.001). The distribution of mothers of controls who were urban dweller was significantly higher than that of cases (70.4 % vs. 51.7 %, *p* = 0.009). The proportion of mothers of controls who had at least primary level of education was significantly higher than that of cases (87.33 % vs. 71.6 %, *p* = 0.001) (Table 3).

**Table 3:** Socio-demographic characteristics of mothers of cases and controls who gave birth at Hawassa public hospitals from March 20-August 30/2018 (*n*=401(134 cases and 267 controls))

Factors	Categories	Fifth minute Apgar score		Total	*p*-value
		Cases N(%)	Controls N(%)		
Age(in years)	<20	12(9)	23(8.6)	35(8.7)	0.768
	20-35	101(75.4)	209(78.3)	310(77.3)	
	>35	21(15.7)	35(13.1)	56(14)	
Residence	Urban	69(51.5)	188(70.4)	257(64.1)	0.009
	Rural	65(48.5)	79(29.6)	144(35.9)	
Educational status	No formal education	38(28.4)	34(12.7)	72(18.0)	0.001
	Primary education	47(35.1)	98(36.7)	145(36.2)	
	Secondary education(9-12)	35(26.1)	86(32.2)	121(30.2)	
	College and above	14(10.4)	49(18.4)	63(15.7)	
Occupation	Housewife	77(57.5)	108(40.4)	185(46.1)	0.01
	Government employee	18(13.4)	56(21.0)	74(18.5)	
	Private employee	31(23.1)	88(33.0)	119(29.7)	
	Others^a^	8(6.0)	15(5.6)	23(5.7)	

Others^a^ student and unemployed

### Obstetrics characteristics of mothers

The proportion of mothers of case who had no ANC contact was significantly no difference with that of controls (8.2 % vs. 3.7 %, *p* = 0.093). The mothers who were physically and emotionally supported during labor and delivery were significantly higher among controls than cases (87.6 % vs. 66.4 %, *P* < 0.001). Antepartum hemorrhage, pregnancy-induced hypertension, and premature rupture of the membrane were more common in cases than controls (11.9–3.7 %, 17.9 to 9.7 %, 20.9 to 18 %) respectively. The difference between controls and cases in antepartum hemorrhage and pregnancy-induced hypertension is statistically significant (Chi-square test, *p*-value = 0.004 and 0.025 respectively). However, the difference is not statistically significant among cases and controls in premature rupture of membrane (chi-square test-*p*-value = 0.501).

Regarding meconium-stained amniotic fluid, the observed difference between cases and controls is statistically significant (33.6 % vs. 15.4 %, *p* < 0.001). In induction of labor, the difference is not statistically significant among cases and controls (20.9 % vs. 17.6 %, *p* = 0.419). Among mothers who were followed for labor and delivery, the prolonged second stage of labor was observed in higher proportion in cases than controls (36.6 % vs. 17.2, *P* < 0.001).

There was no significant difference in the proportion of mothers who were augmented with oxytocin between cases and controls (23.9 vs. 22.8, *p* = 0.803). The proportion of spontaneous vaginal delivery was significantly higher among controls than cases (62.5 % vs. 49.3 %, *P* = 0.039). A hemoglobin level of less than 11 g/dl was observed in 13.4 % of mothers of cases and only 9 % of mothers of controls but the difference is not statistically significant among cases and controls (chi-square test-*p*-value = 0.172). Grand multipara was similar in cases and controls (13.4 % vs. 6.7 %, *p* = 0.498) (Table 4).

**Table 4:** Obstetric characteristics of mothers of cases and controls who gave birth at Hawassa public hospitals from March 20-August 30/2018 (*n*=401(134 cases and 267 controls))

Factors	Category	Fifth minute Apgar score		Total	*p*-value
		Cases N (%)	Controls N(%)		
Physical and Emotional support	Yes	89(66.4)	234(87.6)	323(80.5)	<0.001
	No	45(33.6)	33(12.4)	78(19.5)	
Parity of mother	Primipara	45(33.5)	94(35.2)	139(34.6)	0.498
	Multipara	79(59.0)	159(59.6)	238(59.4)	
	Grand multipara	10(7.5)	14(5.2)	24(6.0)	
ANC contacts	Yes	123(91.8)	257(96.3)	380(94.8)	0.093
	No	11(8.2)	10(3.7)	21(5.2)	
APH	Yes	16(11.9)	10(3.7)	26(6.5)	0.004
	No	118(88.1)	257(96.3)	375(93.5)	
PIH	Yes	24(17.9)	26(9.7)	50(12.5)	0.025
	No	110(82.1)	241(90.3)	351(87.5)	
PROM	Yes	28(20.9)	48(18.0)	76(19.0)	0.501
	No	106(79.1)	219(82.0)	325(81.0)	
Duration of PROM	<8 hrs.	15(53.6)	27(56.2)	42(55.3)	0.821
	≥8 hrs.	13(46.4)	21(43.8)	34(44.7)	
Meconium stained liquor	Yes	45(33.6)	41(15.4)	86(21.4)	<0.00001
	No	89(66.4)	226(84.6)	315(78.6)	
Onset of labor	Spontaneous	106(79.1)	220(82.4)	326(81.3)	0.419
	Induced	28(20.9)	47(17.6)	75(18.7)	
The prolonged second stage of labor	Yes	49(36.6)	46(17.2)	95(23.7)	<0.001
	No	85(63.4)	221(82.8)	306(76.3)	
Augmentation with oxytocin	Yes	32(23.9)	61(22.8)	93(23.2)	0.803
	No	102(76.1)	206(77.2)	308(76.8)	
Mode of delivery	SVD	66(49.3)	167(62.5)	233(58.1)	0.039
	Instrumental delivery	37(27.6)	54(20.2)	91(22.7)	
	Cesarean (C/S)	31(23.1)	46 (17.3)	77(19.2)	
Anemia	Yes	18(13.4)	24(9.0)	42(10.5)	0.172
	No	116 (86.6)	243(91.0)	359(89.5)	
Cord prolapsed	Yes	8(6.0)	3(1.1)	11(2.7)	0.008
	No	126(94.0)	264(98.9)	390(97.3)	

*SVD* Spontaneous Vaginal Delivery

### Fetal/neonatal characteristics

Regarding neonatal sex, males had predominated in both groups (64.9 % among cases and 55.1 % among controls) than females (35.1 and 44.9 %, respectively) and making a ratio of 1.4 but the difference is not statistically significant among cases and controls (chi-square test-*p*-value = 0.068). The proportion of controls that were term was significantly higher than that of cases (90.6 % vs. 82.8 %, *p* = 0.033). Among newborns, 82.1 % of cases were vertex presentation as compared to 91.8 % of controls. Proportions of low birth weight were higher in cases (25.4 % vs. 5.6 %, *p* = 0.012) than that of controls. In the chi-square test, the observed difference between cases and controls in the birth weight is statistically significant at (*p* < 0.001) (Table 5).

**Table 5:** Characteristics of newborns who delivered at Hawassa public hospitals from March 20-August 30/2018 (*n*=401(134 cases and 267 controls))

Factors	Category	Fifth minute Apgar score		Total	*p*-value
		Cases N (%)	Controls N (%)		
Fetal sex	Female	47(35.1)	120(44.9)	167(41.6)	0.068
	Male	87(64.9)	147(55.1)	234(58.4)	
Gestation age at birth	Term	111(82.8)	242(90.6)	353(88.0)	0.033
	Post-term	23(17.2)	25(9.4)	48(12.0)	
Fetal presentation at birth	Vertex	110(82.1)	245(91.8)	355(88.5)	0.012
	Breech	15(11.2)	16(6.0)	31(7.7)	
	Others^a^	9(6.7)	6(2.2)	15(3.7)	
Birth weight	Low birth weight	34(25.4%)	15(5.6%)	34(25.4%)	<0.001
	Macrosomia	8(6.0%)	10(3.7%)	18(4.5%)	
	Normal birth weight	92(68.7%)	242(90.6%)	92(68.7%)	

Others^a^ shoulder, brow, and compound presentation

### Determinant factors of 5th minute low Apgar score

The result on the binary logistic regression revealed that residence, educational level, physical and emotional support during labor and delivery, gravidity of mothers, ANC visit, antepartum hemorrhage (APH), pregnancy-induced hypertension (PIH), meconium-stained amniotic fluid (MSAF), the prolonged second stage of labor, mode of delivery, anemia, fetal sex, gestational age at birth, fetal presentation at birth and fetal weight at birth were significantly associated with the low Apgar score.

In multivariable logistic regression, all significant variables in binary logistic regression were adjusted. The result showed that the odds of 5th minute low Apgar score was about threefold higher among the mothers who lack physical and emotional support during labor and delivery than normal 5th minute Apgar score [AOR = 3.5, 95 % CI: 1.82–6.76]. Rural residence was another variable significantly associated with the low Apgar score. Neonates who delivered with 5th minute low Apgar were four times more likely from rural resident mothers than their counterparts [AOR = 4, 95 % CI: 2.21–7.34]. Fifth minute low Apgar score was 3.5 times more likely on mothers who had no antenatal care contact compared to a normal 5th minute Apgar score [AOR = 3.5, 95 % CI: 1.91–6.33]. Additionally, the chance of low Apgar score was a two-fold increase in newborns who delivered from anemic mothers compared to normal 5th minute Apgar [AOR = 2.3, 95 % CI: 1.10–4.71]. Those newborns who delivered with a 5th minute low Apgar score were six-fold greater odds of having low birth weight compared to their counterparts [AOR = 6.2, 95 % CI: 2.78–14.03] (Table 6). The area under the Apgar score ROC curve was 87.4 %.

**Table 6:** Binary and multivariable logistic regression analysis of determinant factors of low Apgar score among newborns who delivered at Hawassa public hospitals from March - August 2018

Variables	Category	5^th^ minute low Apgar score		Odds ratio (95%CI)	
		Cases	Controls	COR	AOR
Sex	Female	47(35.1)	120(44.9)	1	1
	Male	87(64.9)	147(55.1)	1.5 (0.99-2.32)	1.4 (0.86-2.46)
Gestational age at birth	Term	111(82.8)	242(90.6)	1	1
	Post-term	23(17.2)	25(9.4)	2.0 (1.09-3.69)	0.7 (0.31-1.74)
Fetal presentation	Cephalic	110(82.1)	245(91.8)	1	1
	Breech	15(11.2)	16(6.0)	2.1 (0.99-4.37)	1.5 (0.55-4.01)
	Others^a^	9(6.7)	6(2.2)	3.3 (1.16-9.67)	2.1 (0.57-7.95)
Gestational weight at birth	Low birth weight	34(25.4)	15(5.6)	5.9 (3.10-11.46)	6.2 (2.78-14)**
	Macrosomia	8(6)	10(3.7)	2.1 (0.81-5.50)	2.6 (0.82-8.08)
	Normal	92(68.7)	242(90.6)	1	1
Residence	Urban	69(51.7)	188(70.4)	1	1
	Rural	65(48.5)	79(29.6)	2.2 (1.46-3.44)	4(2.21-7.34)**
Educational status	No formal education	38(28.4)	34(12.7)	3.9 (1.84-8.31)	1.8 (0.64-5.01)
	Primary	47(35.1)	98(36.7)	1.7 (0.84-3.34)	0.9 (0.41-2.17)
	Secondary	35(26.1)	86(32.2)	1.4 (0.70-2.90)	1.1 (0.49-2.47)
	College and above	14(10.4)	49(18.4)	1	1
Physical and Emotional support	Yes	89(66.4)	234(87.6)	1	1
	No	45(33.6)	33(12.4)	3.6 (2.15-5.98)	3.5(1.82-6.76)**
Gravidity	Primigravida	62(46.3)	94(35.2)	1	
	Multigravida	54(40.3)	155(58.1)	0.5 (0.34-0.82)	0.9(0.52-1.56)
	Grand multipara	18(13.4)	18(6.7)	1.5 (0.73-3.14)	0.8(0.33-2.18)
ANC contacts	Yes	123(91.8)	257(96.3)	1	1
	No	11(8.2)	10(3.7)	2.3 (0.95-5.56)	3.5(1.91-6.33)**
APH	Yes	16(11.9)	10(3.7)	3.5 (1.54-7.91)	2.4 (0.84-6.68)
	No	118(88.1)	257(96.3)	1	1
PIH	Yes	24(17.9)	26(9.7)	2.0 (1.11-3.68)	1.4 (0.64-2.93)
	No	110(82.1)	241(90.3)	1	1
MSAF	Yes	45(33.6)	41(15.4)	2.8 (1.71-4.54)	1.2 (0.64-2.22)
	No	89(66.4)	226(84.6)	1	
Prolonged second stage of labor	Yes	49(36.6)	46(17.2)	2.8 (1.72-4.45)	1.3 (0.41-3.91)
	No	85(63.4)	221(82.8)	1	1
Mode of delivery	SVD	66(49.3)	167(62.5)	1	1
	Instrumental	37(27.6)	54(20.2)	1.7 (1.04-2.88)	1.5 (0.81-2.92)
	Cesarean Section	31(23.1)	46 (17.2)	1.7 (0.99-2.92)	0.9 (0.45-1.86)
Anemia	Yes	18(13.4)	24(9.0)	1.571(0.82-3.01)	2.3(1.10-4.71)*
	No	116 (86.6)	243(91.0)	1	1
Others^a^ Shoulder, brow, and compound presentation					
*SVD* Spontaneous Vaginal Delivery					

Key points

*significant at *p*-value <0.05

**significant at *p*-value <0.001

## Discussion

Apgar score can be influenced by various factors that occur before and during pregnancy including the physical and emotional support during labor and delivery. Therefore, this study identified the determinant factors for a low Apgar score which is important for proper, immediate, and sustainable intervention to improve maternal for better pregnancy outcomes.

This study revealed that the odd of lack of physical and emotional support during labor and delivery was higher among 5th minute low Apgar score newborns than normal 5th minute Apgar score. This finding is consistent with a study done in New York City [21]. This similarity could be due to that physical and emotional support during labor and delivery reduces the need for medical intervention, cesarean rate, length of labor, and increase the spontaneous labor [22], which could further reduce the Apgar score.

This study showed that neonates who delivered with 5th minute low Apgar were more likely from rural resident mothers than their counterparts. This study is similar to the study done in Australia and Gondar [11, 23]. This could be due to the health-seeking behavior of rural communities is low and access to health care for pregnant women may be more difficult, which could complicates fetal outcome. This is supported by the studies conducted in the developed countries in which the residence is not risk factors of low Apgar score [19, 24, 25]. Lack of transport, distance, difficult topography, the cost of ambulance fuel, use of an ambulance for unintended purpose and uncooperative behavior of ambulance drivers were detected in the study area by other studies [26].

Fifth minute low Apgar score was more likely on newborns who delivered from anemic mothers compared to normal 5th minute Apgar score. This study is consistent with studies done in Sweden, Brazil, the USA, and Gondar [11, 19, 24, 25]. This similarity is due to the fact that, anemia during pregnancy puts infants at increased risk of immediate birth asphyxia and long-term permanent cognitive damage and mortality [27]. Anemia in pregnancy is more prevalent in the developing world, where under nutrition and infections are more frequent which complicates pregnancy outcome. However, a study done in Amhara region Ethiopia and Japan indicated that anemia during pregnancy is not a determinant factor for birth asphyxia/low Apgar score [17, 28]. This difference could be due to high prevalence of a history of antepartum hemorrhage in this study and difference in study setting. This finding suggests that health care providers should take into account the potential risk of anemia during pregnancy while assessing clinical condition of the mothers.

The risk of the low Apgar score was higher in newborns whose mothers had no antenatal care contact compared to normal 5th minute Apgar. This finding is similar to studies done in Sweden [25, 29]. This could be the fact that supplementation, treatments, birth preparedness and readiness and counseling for a danger sign of pregnancy is given during antenatal care follow-up. Medical care for women with obstetric complications begins with the recognition of danger signs. Delays in recognizing danger signs, in seeking, reaching, and obtaining appropriate maternity care are key elements in maternal and perinatal health [30]. The other possibility could be because mothers who had no ANC visits will not gain a screening opportunity for certain risk factors like, anemia during pregnancy and low birth weight which were the risk factors for the low Apgar score in this study. This finding also indicates that the antenatal care follow up efficiently will help to mitigate the problem of low Apgar score.

Low birth weight was a major determinant factor that was significantly associated with a low Apgar score in the present study. Those newborns who delivered with a 5th minute low Apgar score were higher odds of having low birth weight compared to their counterparts. This finding is also consistent with the studies conducted in Ethiopia, Iran, and Ghana [11, 18, 31]. This could be explained by the fact that small babies might suffer from difficult birthing, impaired thermoregulation, hypoglycemia, polycythemia and might develop difficulty in cardiopulmonary transition and perinatal asphyxia, which could further affect Apgar score [32–34].

## Conclusion and recommendation

In the current study lack of physical and emotional support during labor and delivery, rural residence, lack of ANC follow-up, Anemia during pregnancy, and low birth weight were determinant factors of a low Apgar score. In addition to the standard care of using electronic fetal monitoring, increasing access to compassion ship during labor and delivery was recommended. Effective health educations especially in promoting healthy wellbeing during preconception and ANC care will help in detecting high-risk pregnancy that leads to a low Apgar score. Health professionals should screen and consulate pregnant mothers who are at risk of having infants with low Apgar scores and ensure that women have access to the essential health information on the determinants of a low Apgar score. Public education and awareness on how to carry on a healthy pregnancy should be given. Likewise, women should be linked to the appropriate maternal health services including antenatal care and nutritional counseling services. Further studies, preferably prospective cohort studies that include evaluation of individual-level behavioral and psychological factors, environmental exposures, medical conditions, biological factors, and genetics characteristics are needed to identify and characterize the underlying causes of a low Apgar score in Hawassa city and elsewhere.

## Limitation of study

Information pertaining to several determinant factors of low Apgar score previously reported, such as analgesia during labor and delivery, uterine rupture, fetal heart rate abnormalities, diabetes mellitus, an antipsychotic drug, thrombocytopenia, and thyroid diseases were not available for the study. The study was restricted to the term and post-term pregnancies and the results may not be generalizable to preterm, severe congenital malformation, and multiple pregnancies. The outcome variable was > 10 %, the odds ratio used in the study might be overestimated.

Generally does not allow calculation of incidence (absolute risk), inefficient for rare exposures, and information on exposure was subject to observation bias. Due to the study design, there was intra-observer variability.

## Data Availability

All related data has been presented within the manuscript. The data set supporting the conclusions of this article is available from the authors on request.

## Abbreviations

ANC: Antenatal Care
AOR: Adjusted Odds Ratio
APGAR: Appearance Pulse Grimace Activity Respiration
APH: Antepartum hemorrhage
CI: Confidence interval
HUCSH: Hawassa University Comprehensive Specialized Hospital
MSAF: Meconium Stained Amniotic Fluid
MSAF: Meconium Stained Amniotic Fluid
PIH: Pregnancy Induced Hypertension
SVD: Spontaneous Vaginal Delivery
